# Superoxide solution irrigation for septic arthritis: a prospective clinical study of efficacy and a widened therapeutic window

**DOI:** 10.3389/fmed.2026.1897673

**Published:** 2026-08-28

**Authors:** Badrul Akmal Hisham Md. Yusoff, Muhammad Farhan Md. Yusoff, Norlelawati Mohamad, Muhamad Karbela Reza Ramlan, Nik Kamarul Arif Nik Kamrulzaman, Azwan Aziz Mohamad

**Affiliations:** Department of Orthopaedic & Traumatology, Faculty of Medicine, Universiti Kebangsaan Malaysia, Kuala Lumpur, Malaysia

**Keywords:** arthrotomy therapeutic irrigation, biofilms, infection, knee joint, septic arthritis

## Abstract

**Background:**

Septic arthritis is an orthopedic emergency, with the knee being the most commonly affected joint. Standard irrigation with normal saline does not provide biofilm-disrupting efficacy, a key factor in treatment failure, especially against virulent organisms such as *Staphylococcus aureus* and *Pseudomonas aeruginosa*. This study evaluates the use of Hydrocyn, a superoxidized solution with known antibiofilm properties, as an intraoperative joint irrigant.

**Methods:**

A prospective case series was conducted on 17 adults with native knee septic arthritis from 1 February 2024 to 31 December 2024. All patients underwent standard arthrotomy or arthroscopic washout with joint irrigation using Hydrocyn. Primary outcomes included serial measurement of inflammatory markers, including C-reactive protein (CRP) levels and white blood cell count (WBC), as well as knee range of motion (ROM) at baseline, 1, 2, and 6 weeks; ROM was further assessed up to 12 weeks postoperatively.

**Results:**

Pathogens were identified in 94.1% of cases, including methicillin-resistant *Staphylococcus aureus* (MRSA) (17.6%) and *P. aeruginosa* (11.8%). Significant improvements were observed in CRP levels (*p* < 0.001), WBC (*p* = 0.001), and knee ROM (*p* < 0.001) at 6 weeks. The reoperation rate due to reinfection was 5.8%. Patients undergoing irrigation more than 24 h after admission demonstrated improvements in outcomes equivalent to those treated within 24 h, with no statistical difference between the groups.

**Conclusion:**

Superoxide solution irrigation, combined with antibiotics, surgical debridement, and physiotherapy, is associated with excellent clinical outcomes, including rapid normalization of inflammatory markers, functional recovery, and a low reoperation rate. Its potent biofilm-disrupting action may mitigate the adverse effects of delayed surgical intervention, challenging current treatment paradigms.

## 1 Introduction

Septic arthritis is a serious joint infection primarily caused by bacterial pathogens (1, 2). The knee is the most commonly affected joint, surpassing other anatomical sites in prevalence (3). Clinically, septic arthritis can be categorized as acute (duration <3 weeks) or chronic (duration >3 weeks), both of which fall under the broader definition of septic arthritis (4). Due to its potential for rapid joint destruction, septic arthritis constitutes a surgical emergency, necessitating urgent evaluation, accurate diagnosis, and immediate intervention. Early recognition and treatment are critical to minimizing complications and reducing mortality rates. Conversely, delayed or inadequate management may lead to irreversible joint damage, long-term disability, and, in severe cases, fatal outcomes (3, 5). When infection is confirmed or strongly suspected, prompt arthroscopic joint irrigation and debridement, including synovectomy, should be performed to mitigate disease progression. This procedure removes toxins and reduces both the bacterial load and intra-articular pressure.

Previously, diluted iodine was used for joint irrigation during arthrotomy washout. Povidone-iodine is bactericidal against Gram-positive and Gram-negative organisms and has been used to treat septic arthritis. In a preclinical study on the effect of povidone-iodine on cartilage, bovine cartilage explants (*n* = 42) were exposed to varying iodine concentrations (0.35% and higher doses) for 1, 3, or 6 min, with saline as the control (6). A live/dead cytotoxicity assay revealed dose- and time-dependent chondrotoxicity, with significant superficial chondrocyte death observed at higher concentrations and prolonged exposure. While 0.35% iodine demonstrated the least toxicity, even a brief application for >1 min significantly impaired cell viability (6). These findings suggest that iodine irrigation, regardless of concentration, exerts marked chondrotoxic effects on superficial cartilage after exposures exceeding 1 min, warranting caution in clinical practice, especially during arthrotomy washout (7). Current practice uses only normal saline as a joint irrigation solution during arthrotomy washout.

Biofilms play a significant role in the development and persistence of septic arthritis, a bacterial infection of the joints, contributing to its treatment resistance and increased risk of recurrence. Bacteria such as *Staphylococcus aureus* and *Pseudomonas aeruginosa* can form biofilms in the synovial fluid of infected joints, shielding themselves from the host’s immune system and antibiotics (8). Consequently, *Staphylococcus aureus* is associated with high rates of treatment failure. Compounding this clinical challenge is the increasing prevalence of methicillin-resistant *Staphylococcus aureus* (MRSA) in septic arthritis, which exhibits higher mortality rates than infections caused by methicillin-susceptible *Staphylococcus aureus* (MSSA). Although normal saline is useful for wound irrigation, it is generally ineffective at removing or disrupting bacterial biofilms, which are complex microbial communities encased in a self-produced matrix (9). It can help mechanically remove some bacteria and debris but it does not penetrate or break down the biofilm matrix. Various adjunctive therapies, such as phage therapy and immunomodulators, have shown promising results but are not cost-effective (8).

In contrast, superoxidized solutions are widely used for wound cleansing and are effective against biofilms. Superoxidized solution comprises pure neutral-pH water and sodium chloride and is produced through electrolysis. The process produces reactive oxygen species, causing protein denaturation and producing a bactericidal effect without damaging the host tissue (10, 11). The inadequacy of saline against biofilms and the chondrotoxicity of iodine make superoxide solution a potential alternative. Preclinical studies have shown that superoxide solution is non-toxic to osteoblasts and chondrocytes (12, 13). This pilot feasibility study aimed to evaluate the clinical outcomes of superoxide solution as an intraoperative joint irrigant during arthrotomy washout in native joint septic arthritis.

## 2 Methods

### 2.1 Study design

This prospective case series was conducted at a single center and included 17 patients with native knee septic arthritis who were referred to us from 1 February 2024 to 31 December 2024. We included patients older than 18 years with a first episode of native knee septic arthritis. We excluded patients with suspected acute gouty arthritis, prosthetic joint infection, preexisting septic arthritis of the knee, or recurrent septic arthritis. We also excluded patients with immunosuppressive conditions such as HIV infection, those receiving active chemotherapy, or those taking immunosuppressive medications, with the exception of uncomplicated diabetes mellitus. This study was conducted in accordance with the ethical standards of the institution and approved by the UKM Ethical Committee (JEP-2023-909). Before treatment began, written informed consent was obtained from all subjects involved in the study. All procedures were performed in compliance with the ethical standards set forth in the Declaration of Helsinki. All patients agreed to participate in this study and provided written consent; no patient requested conventional irrigation. Because this was a pilot feasibility study, prospective clinical trial registration was not undertaken, consistent with standard practice for pilot studies at our institution.

The diagnosis was made based on (i) history and physical examination, including fever, presence of injury or direct inoculation, inability to weight-bear, painful range of motion, and (ii) septic parameters such as increased white blood cell count (WBC) and raised C-reactive protein (CRP), and (iv) bacteria seen in synovial fluid analysis (5). All findings were recorded in the electronic medical record. Patients received standard care from the primary team, including early and appropriate initiation of broad-spectrum antibiotics, supportive care, analgesia, and admission to the wards.

**Figure 1:**
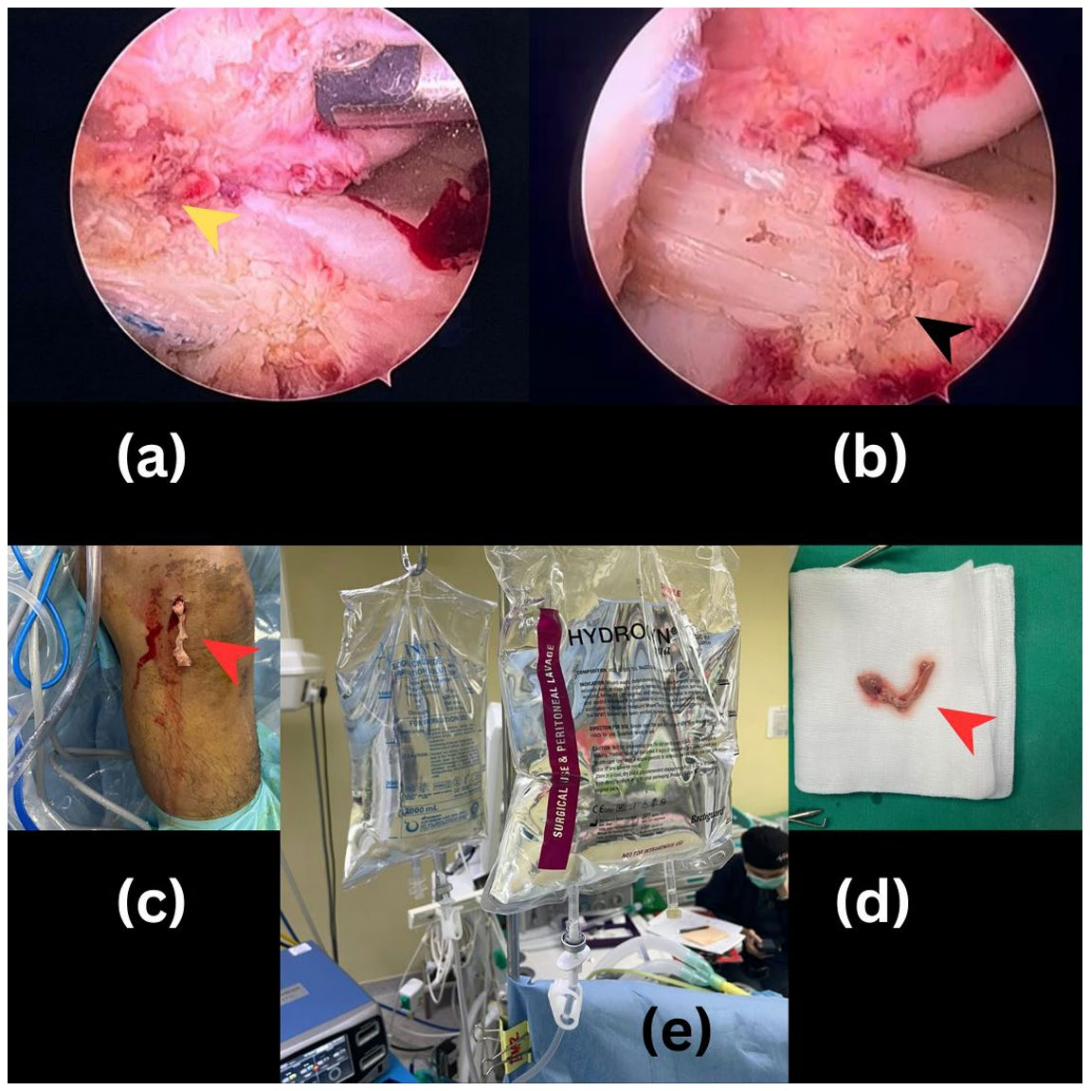
Showed the steps for protocol using Hydrocyn. This image showed the arthroscopic washout of right knee for septic arthritis post anterior cruciate ligament reconstruction. Note the slough intra-articular (yellow arrow) in image **(a)**, and slough at the graft (black arrow head) **(b)**. Then, the slough (red arrow head) is debrided and removed (image **c** and **d**). Then, the joint is irrigated using normal saline until the colour is clear, less turbid and no debris. This is followed by hydrocyn irrigation of 1L, and it is kept inside the knee for 1 min (image e). Then, Hydrocyn is removed and re-irrigated with 1 L of normal saline. Then, wound is sutured.

Fourteen patients underwent open knee arthrotomy washout, and three patients underwent arthroscopic washout, according to standard practice, except that joint irrigation was performed using the superoxide irrigation protocol as shown in Figure 1. All patients underwent either open arthrotomy or arthroscopic washout under standard sterile conditions. Following joint exposure and macroscopic assessment of the intra-articular contents, synovial fluid, purulent material, and synovial tissue samples were collected intraoperatively and sent for microbiological culture and antimicrobial susceptibility testing *prior* to irrigation. Following sample collection, the joint was irrigated with normal saline (0.9% sodium chloride) until the effluent was macroscopically clear, with a visible reduction in turbidity and complete removal of particulate debris and fibrinous material. Subsequently, 1 L of Hydrocyn, a superoxidized solution containing 0.003% hypochlorous acid (HOCl) at a concentration of 30 parts per million (ppm), was administered either via arthroscopic lavage or by manual instillation into the joint space. Hydrocyn was administered as an off-label intraoperative irrigant with ethics committee approval. The solution was allowed to dwell intra-articularly for 1–2 min without the application of predetermined irrigation pressure. Following the dwell time, the joint was irrigated again with 1 L of normal saline to remove any residual superoxidized solution. The surgical wound was then closed in layers, and a sterile dressing was applied.

Postoperatively, patients received standard postoperative care, including monitoring of vital signs, pain scores, and range of motion. All patients were referred to inpatient physiotherapy. Antibiotics were adjusted by either the primary team or the infectious disease team according to the culture and sensitivity report. Septic parameters, such as WBC and CRP, were monitored at 1-week, 2-week, and 6-week postoperatively, and range of motion was assessed up to 12 weeks postoperatively. One patient was admitted to the intensive care unit during the study period. Upon discharge, patients received standard discharge care, including a medical certificate, analgesia, a home exercise program, and outpatient physiotherapy.

### 2.2 Statistical analysis

Continuous variables were presented as mean ± standard deviation (SD) for normally distributed data. Categorical variables were reported as frequencies and percentages. For the primary analysis of clinical outcomes over time (CRP, WBC, and knee flexion ROM), a repeated-measures analysis of variance (ANOVA) was performed on the full cohort of 17 patients, with each patient serving as their own control.

Subgroup comparisons, including those based on timing of surgery (<24 h vs. >24 h) (5), were exploratory in nature due to the limited sample sizes. For these analyses, the non-parametric Mann–Whitney U-test was used. No adjustments were made for multiple comparisons, and these results should be interpreted with caution. All statistical analyses were performed using SPSS version 26.0 (IBM Corp., Armonk, NY, USA). A *p*-value of < 0.05 was considered statistically significant for the primary analysis, while exploratory subgroup findings were presented as hypothesis-generating.

## 3 Results

A total of 17 patients with native knee septic arthritis were included in this study. The cohort had a mean age of 51.4 ± 17.6 years and predominantly comprised men (70.6%, *n* = 12), with women accounting for 29.4% (*n* = 5). Comorbidities were present in 11 patients: two had concurrent chronic kidney disease and diabetes, eight had diabetes alone, and one had chronic kidney disease alone; the remaining patients had no significant comorbidities (Table 1). The mean HbA1c was 6.7 ± 1.2, and the mean hemoglobin level was 12.1 ± 1.5. The suspected etiology of infection was spontaneous in 47.1% (*n* = 8) of cases, followed by local spread (23.5%, *n* = 4), direct inoculation (17.6%, *n* = 3), and bacteremia (11.8%, *n* = 2). Fourteen patients underwent open knee arthrotomy washout, and three patients underwent arthroscopic washout.

**Table 1:** Baseline demographic data for 17 patients with septic arthritis.

Demographic data		Value
Age, years		51.4 (17.6)*
Gender	Male	12 (70.6)^
	Female	5 (29.4)^
Comorbidities	Diabetes	8 (47.0)^
	Chronic kidney disease	1 (5.8)^
	Diabetes with chronic kidney disease	2 (11.7)^
Baseline blood investigation	HbA1c, %	6.7 (1.2)*
	Hemoglobin, g/dL	12.1 (1.5)*

*Expressed as mean (standard deviation).

^Expressed as frequency and percentage.

Microbiological analysis of synovial fluid cultures identified a pathogen in 16 cases (94.1%). The most common isolates were from the *Staphylococcus aureus* group (41.1%, *n* = 7), comprising methicillin-sensitive *S. aureus* (*n* = 4) and methicillin-resistant *S. aureus* (*n* = 3). *Streptococcus* species were isolated in 29.4% (*n* = 5) of cases, including *Streptococcus pyogenes* (*n* = 1), *Streptococcus dysgalactiae* (*n* = 2), and *Streptococcus agalactiae* (*n* = 2). Gram-negative bacteria accounted for 23.5% (*n* = 4) of isolates, specifically *P. aeruginosa* (*n* = 2), *Achromobacter* species (*n* = 1), and *Klebsiella pneumoniae* (*n* = 1). The remaining cultures showed no growth (Table 2).

**Table 2:** Etiological organisms identified in patients with septic arthritis.

Pathogen group	Pathogen species	*N* (%)
*Staphylococcus aureus* group		7 (41.1)
	Methicillin-sensitive *Staphylococcus aureus*	4 (23.5)
	Methicillin-resistant *Staphylococcus aureus*	3 (17.6)
*Streptococcus* species		5 (29.4)
	*Streptococcus pyogenes*	1 (5.8)
	*Streptococcus dysgalactiae*	2 (11.7)
	*Streptococcus agalactiae*	2 (11.7)
Gram-negative bacteria		4 (23.5)
	*Pseudomonas aeruginosa*	2 (11.7)
	*Achromobacter* species	1 (5.8)
	*Klebsiella pneumoniae*	1 (5.8)
Culture-negative		1 (5.8)

The mean hospital stay was 16.7 ± 5.1 days. The majority of patients were admitted to the general ward. One patient with severe sepsis and a negative synovial culture died of hospital-acquired pneumonia. One patient required a second arthrotomy washout during admission because of *P. aeruginosa* infection on day 7; the patient subsequently recovered. However, repeated synovial fluid cultures showed no bacterial growth. The reoperation rate due to reinfection was 6.25% (one of 16).

A total of 16 patients completed 6 weeks of follow-up. All patients showed significant improvements in CRP (*p* < 0.001), WBC (*p* = 0.001), and knee ROM (*p* < 0.001) at 6 weeks compared with baseline, as shown in Table 3. At 6 weeks, the mean flexion range of motion was 105 ± 10.9 degrees and improved further at 3-month follow-up (118.7 ± 8.6 degrees; *p* < 0.001).

**Table 3:** Clinical outcomes of superoxide solution irrigation up to 12-week postoperatively.

Clinical outcomes	Preoperative (*n* = 17)	Postoperative week 1 (*n* = 16)	Postoperative week 2 (*n* = 16)	Postoperative week 6 (*n* = 16)	Postoperative week 12 (*n* = 16)	*p*-value	Mean difference	95% CI
CRP, mg/dL	18.9 (10.9)	9.2 (6.8)	4.0 (3.3)	1.6 (2.1)	–	<0.001	17.3	9.9–24.9
WBC, thousand/μL	13.1 (7.6)	10.0 (4.3)	9.1 (3.5)	7.9 (1.5)	–	0.001	5.2	0.05–10.4
Flexion ROM, degrees	50.6 (31.8)	84.6 (19.6)	93.1 (10.1)	105.1 (10.9)	118.7 (8.6)	<0.001	−67.5	−96.27 to −38.72

Repeated-measures ANOVA was used to assess clinical outcomes at 1-week, 2-week, 6-week, and 12-week postoperatively; a *p*-value < 0.05 was considered statistically significant.

CRP, C-reactive protein; WBC, white blood cell count; ROM, range of motion; CI, confidence interval.

Six patients (37.5%) underwent washout after 24 h of admission, while 10 patients (62.5%) underwent washout within 24 h of admission. A comparison of patients who underwent washout within or after 24 h of admission demonstrated marked reductions in CRP and normalization of WBC in both groups. At 6 weeks, both groups showed improvements in CRP and knee range of motion, as well as normalization of WBC (Table 4).

**Table 4:** Comparison of clinical outcomes after superoxide solution irrigation between patients who underwent knee washout within 24 h and after 24 h.

Timing of surgery	Preoperative			Postoperative week 1			Postoperative week 2			Postoperative week 6		
	<24 h *n* = 10	>24 h *n* = 6	*p*-value	<24 h *n* = 10	>24 h *n* = 6	*p*-value	<24 h *n* = 10	>24 h *n* = 6	*p*-value	<24 h *n* = 10	>24 h *n* = 6	*p*-value
CRP, mg/dL	20.1 (11.1)	12.2 (11.7)	0.079	8.3 (11.7)	6.9 (9.5)	0.257	3.4 (2.2)	2.6 (5.2)	0.910	1.1 (0.7)	1.5 (2.2)	0.729
WBC, thousand/μL	11.0 (6.0)	14.0 (6.0)	0.425	11.0 (6.0)	9.0 (4.0)	0.424	8.0 (3.0)	8.0 (2.0)	0.770	8.0 (3.0)	7.0 (2.0)	0.108
Flexion ROM, degrees	45 (80)	50.0 (60)	0.486	90 (10)	90 (47.5)	0.058	90 (11.0)	90 (10)	0.945	110 (10)	110 (25)	0.443

The Mann–Whitney U-test was used to compare the clinical outcomes at different time points at 1-week, 2-week, 6-week, and 12-week postoperatively.

CRP, C-reactive protein; WBC, white blood cell count; ROM, range of motion.

## 4 Discussion

This study is the first to investigate the efficacy of a superoxide solution as an irrigant in the management of knee septic arthritis, demonstrating promising results compared with the historical standard of normal saline. The objective of arthrotomy washout is to remove toxins and reduce both the bacterial load and intra-articular pressure (1, 14).

The findings are contextualized by prior research. Interestingly, superoxide solution was associated with a lower reinfection rate requiring a second arthrotomy washout (*n* = 1, 5.8%) despite 41.2% of patients undergoing surgery more than 24 h after admission, compared with Balabaud et al. (15) (*n* = 15, 60%), Peres et al. (16) (*n* = 2, 18.2%), Johns et al. (17) (*n* = 90, 55.9%), Bovonratwet et al. (18) (*n* = 24, 14.2%), and Bohler et al. (19) (*n* = 6, 20.7%). Furthermore, the reduction in CRP within 1 week (51.3%) was comparable to that reported by Peres et al. (16) (72.9% with arthrotomy saline washout and 66.3% with arthroscopic saline washout, respectively). While this is comparable to the reduction observed in our study (51.3%), a notably higher rate of negative synovial cultures (52.4%) was reported in their study, which may indicate a positive treatment response, though it could also reflect limitations in culture sensitivity.

Our patients had longer hospital stays than those reported by Bovonratwet et al. (18) (8.1 days) and Dave et al. (20) (10.9 days). This was because most patients required completion of 2 weeks of intravenous antibiotics. The mortality rate in our study (1 of 16, 6.25%) due to severe sepsis was comparable to that in other studies: Faour et al. (21) (two of 231, 0.8%), Bovonratwet et al. (18) (2 of 168, 1.19%), and Johns et al. (17) (9.5%).

A particularly surprising finding was that patients who underwent irrigation more than 24 h after admission demonstrated improvements in CRP, WBC, and ROM equivalent to those treated within 24 h. This challenges the long-held view that immediate surgical intervention is the sole determinant of outcome in septic arthritis. While expedient treatment remains paramount, the use of a highly effective biofilm-disrupting irrigant such as superoxide solution may widen the therapeutic window for surgical management (22). This could be invaluable in real-world clinical settings, where operating room availability or patient transfer delays are inevitable. This finding warrants further investigation in larger, randomized controlled trials. Unlike normal saline, which acts merely as a mechanical irrigant, superoxide solution delivers reactive oxygen species (ROS) directly to the biofilm-protected nidus of infection (23–25). ROS are known to cause oxidative damage to bacterial cell membranes and proteins, effectively penetrating and disrupting the extracellular polymeric substance of biofilms (11). This biofilm-disrupting capability is critical in treating virulent organisms such as *S. aureus* and *P. aeruginosa*, which are notorious for biofilm formation and treatment resistance.

This case series has several strengths, primarily its novelty as the first investigation into the efficacy of a superoxide solution for joint irrigation in native knee septic arthritis, directly addressing a significant unmet need in managing biofilm-associated infections. The prospective design ensured rigorous and standardized data collection, enhancing the reliability of the findings. Furthermore, comprehensive microbiological profiling, which captured a spectrum of virulent and biofilm-forming pathogens, including MRSA and *P. aeruginosa*, strengthens the clinical relevance and applicability of the results. The objective measurement of outcomes through serial inflammatory markers and functional range-of-motion assessments provides robust, quantitative evidence of patient improvement. The inclusion of a heterogeneous patient population with various comorbidities and infection etiologies supports the potential generalizability of these findings to broader clinical practice. However, this study is subject to several limitations. The main limitation is the absence of a concurrent control group, which makes it challenging to definitively attribute the observed outcomes solely to the superoxide solution irrigant rather than to other components of standard care, despite comparisons with historical data. The small sample size limits the statistical power of the analysis and precludes meaningful subgroup analyses of factors such as pathogen-specific responses. As a single-center study, there is an inherent risk of selection bias, and the results may not be fully generalizable to other institutions with different patient demographics or surgical protocols. Additionally, while short-term outcomes at 6 weeks and 3 months are encouraging, a longer follow-up period would be necessary to comprehensively assess functional recovery, the long-term risk of post-infectious osteoarthritis, and the definitive rate of infection recurrence.

## 5 Conclusion

In conclusion, this case series demonstrates that the use of Hydrocyn, a superoxide solution, as an intraoperative joint irrigant for native knee septic arthritis was safe and associated with highly promising clinical outcomes. The observed rapid normalization of inflammatory markers, significant functional improvement, and a notably low reoperation rate of 5.8% suggest a potential therapeutic advantage over traditional normal saline irrigation. This efficacy is likely attributable to the solution’s unique biofilm-disrupting mechanism of action, which appears to be particularly effective against virulent organisms such as *S. aureus* and *P. aeruginosa*. However, further randomized controlled trials are needed to determine the superiority of superoxide solution over currently used joint irrigants.

Perhaps the most striking finding was that irrigation with superoxide solution may mitigate the adverse consequences of delayed surgical intervention, a discovery that could challenge existing treatment paradigms and prove invaluable in real-world clinical settings. The promising results presented here, especially when contrasted with historical data on saline irrigation, strongly support a paradigm shift in the standard surgical management of this orthopedic emergency. Therefore, we assert that these findings warrant validation through large-scale, multicenter, randomized controlled trials. If confirmed, superoxide solution irrigation has the potential to establish a new evidence-based standard of care, ultimately improving outcomes and reducing the burden of this devastating disease.

## Data Availability

The datasets presented in this study can be found in online repositories. The names of the repository/repositories and accession number(s) can be found in the article/supplementary material.
